# Variable Importance Measures Suggest Paramount Influence of Human Economics on Alien‐Species Introductions

**DOI:** 10.1002/ece3.70965

**Published:** 2025-02-12

**Authors:** Ignasi Arranz, Ralph Mac Nally, Emili García‐Berthou

**Affiliations:** ^1^ Instituto De Investigación En Cambio Global (IICG‐URJC) Universidad Rey Juan Carlos Móstoles Spain; ^2^ Departamento De Biología y Geología, Física y Química Inorgánica Universidad Rey Juan Carlos (URJC) Móstoles Spain; ^3^ Centre For Applied Water Science And Institute For Applied Ecology University Of Canberra Canberra Australian Capital Territory Australia; ^4^ School Of Biosciences The University Of Melbourne Parkville Victoria Australia; ^5^ GRECO, Institute Of Aquatic Ecology University Of Girona Girona Catalonia Spain

**Keywords:** global environmental change, invasive non‐native species, machine learning, multiple regression analysis, variable importance

## Abstract

Identifying the most important variables that determine patterns and processes is one of the main goals in many scientific fields, including ecological and evolutionary studies. Variable or relative importance is generally seen as the proportion of the variation in a response variable explained directly and indirectly by a specific predictor. Although partial regression coefficients are perhaps the most frequently used, ‘standard’, statistical technique in ecological and evolutionary studies, they are inadequate indices of variable importance when predictors are intercorrelated, which tends to be the rule in most observational data sets. Among other statistical techniques, random forests and hierarchical partitioning are designed to cope with collinearity but are still much less used than beta weights to measure variable importance. Here, we compared random forests and hierarchical partitioning with linear mixed models to attempt to unravel the individual and shared variation of environmental, economic, and human population factors with success of alien species richness in eight taxonomic groups at a global scale. Results showed that random forests and hierarchical partitioning generally agreed in ranking variable importance but showed considerably different conclusions to the standard statistical approach. Specifically, random forests and hierarchical partitioning attached more importance to economic and human population variables in explaining spatial patterns of alien species richness than did region area and mean air temperature, which were emphasized more by the standard approach. Beta weights also tended to highlight less correlated predictors, such as sampling effort and precipitation. Variable importance in random forests attached more importance to economic than population variables and to absolute rather than relative predictors. In conclusion, using variable importance measures enable to better identify the most significant drivers of biological invasions but it can also be applied to other biological and scientific questions, leading to tackle more efficient management and conservation decisions in global change research.

## Graphical abstract

The current paper compares several statistical techniques to estimate the variable importance of the main drivers shaping the global distribution of alien species richness in eight taxonomic groups. Variable importance measures can be applied to address other biodiversity questions, particularly those arising from the impacts of global change.

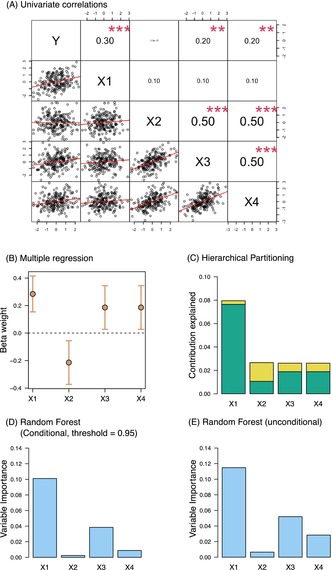

## 1 Introduction

Identifying the factors that are most likely to explain variation in a response variable or phenomenon is a primary goal of science, including in the fields of ecology and evolution. Examples include investigating the relative importance of abiotic and biotic factors for species distribution and coexistence (Gravel, Guichard, and Hochberg 2011; Harisena et al. 2021) or identifying best practices for conservation success (Kapos et al. 2008). However, the importance of different predictors in determining a relevant response variable is poorly understood because, for instance, species distributions generally do not respond to a single predictor and many environmental factors are correlated. Further, in a complex world, not all variables of importance are known or can be anticipated, while only proxy data may be available (e.g., economic activity and human population used as proxies of invasion propagule pressure and environmental disturbance), particularly if analyses cover large spatial and temporal extents (e.g., global phenomena).

There is no universal definition of variable or relative importance (Achen 1982; Budescu 1993; Grömping 2015; Wei, Lu, and Song 2015), but it can be viewed as the proportion of the variation of the response variable potentially explained directly and indirectly by a given predictor (Johnson and Lebreton 2004). Traditionally, multiple linear regression, its univariate generalizations (e.g., generalized linear and mixed models), or multivariate equivalents (e.g., redundancy analysis) have been used for modeling and identifying the likely most important predictors from a given data set (Weisberg 1985; Montgomery, Peck, and Vining 2006). These methods are reasonably robust for prediction (Houlahan et al. 2017), theory‐testing goals (Johnson and Lebreton 2004), and for pattern identification (Grömping 2009; Nathans, Oswald, and Nimon 2012). Partial regression coefficients or their standardized version (i.e., beta weights) are probably the most widely used measures to draw conclusions about the relative importance of predictors in ecology and evolution (Table S1) (Darlington and Hayes 2017). Beta weights are the change (in standard deviation units) in the response variable associated with a change by one standard deviation in a given predictor if other predictors are held constant (Weisberg 1985; Montgomery, Peck, and Vining 2006). When predictors are completely uncorrelated, zero‐order correlations and standardized regression coefficients are equivalent, so the relative importance of predictors can be expressed as the proportion of predictable variance for which it accounts.

**FIGURE 1:**
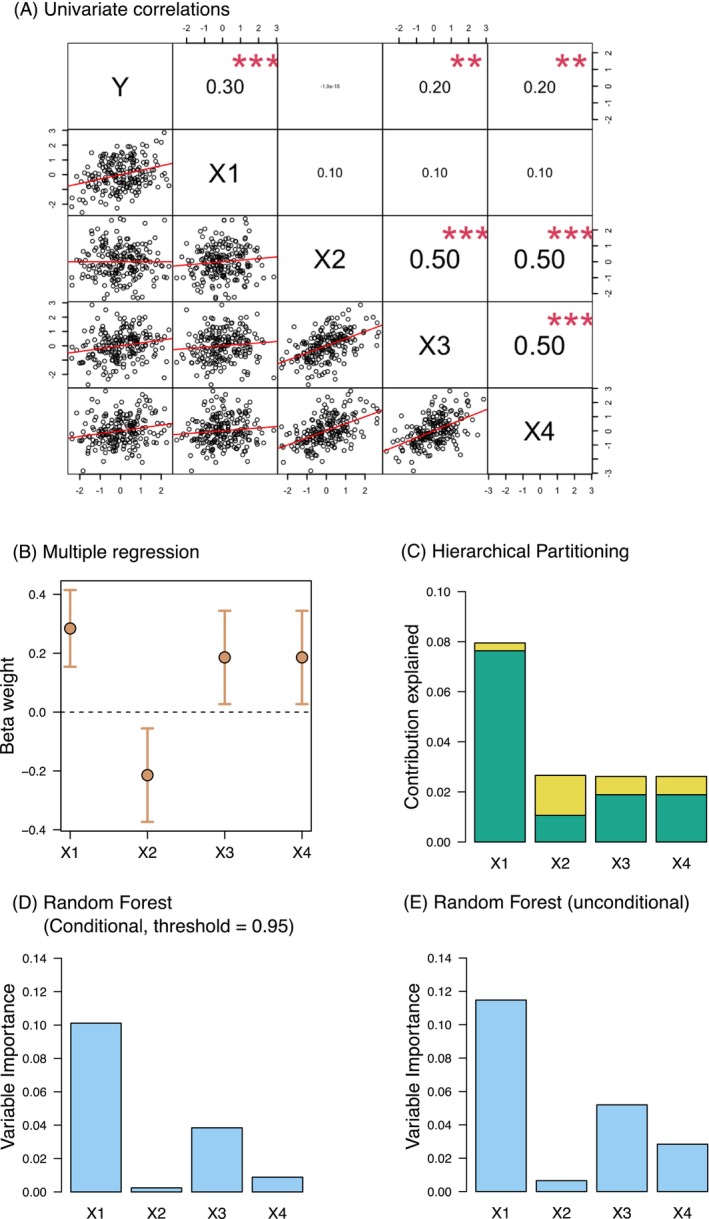
Comparative assessment of statistical methods to quantify variable importance with simulated data (see script in Figure S1). When one variable (X2 in Panel A) is strongly correlated with the other two (X3 and X4 in Panel A), the beta weights from the multiple regression approach (Panel B) may overestimate the variable importance of X2. Hierarchical partitioning (Panel C) and Random forests analyses (Panels D and E) can cope with the multicollinearity among variables and the variable importance of X2 decreases. Simulated data were modified from Ray‐Mukherjee et al. (2014). **, *p* < 0.01; ***, *p* < 0.001.

Beta weights are inadequate indices of variable importance when predictors are correlated, especially when correlations are high (Hoffman 1960; Budescu 1993; Green and Tull 1974; Grömping 2015; Wei, Lu, and Song 2015), which typifies empirical studies because many potential predictors covary (Azen and Budescu 2003). When two or more predictors in a model are not statistically independent of one another, the reliability of beta weights is lessened (Farrar and Glauber 1967). Although there are many rules of thumb for addressing multicollinearity (e.g., excluding variables that have *r* ≥ |0.70| with one or more other variables), the more variables included in the model, the greater is the potential for multicollinearity or association among variables (Strobl et al. 2008) and the identification of the actually influential variables becomes more problematic. Moreover, beta weights depend not only on the effect size of the predictor but also on its variance (and thus the sampling range or design), among other factors (Greenland et al. 1991). The characterization of variable importance becomes a challenging task for which the outputs from linear regression models are not well suited (Grömping 2007). Although reliance on beta weights is a common practice in many fields including ecology and evolution (Table S1), beta weights provide limited information (Nathans, Oswald, and Nimon 2012). A hypothetical example (obtained with the script in Figure S1 and similar to other ones in the statistical literature) that illustrates these points is presented in Figure 1.

Many methods have been developed in the last few decades to attempt to quantify the relative importance of intercorrelated predictors (Liakhovitski, Bryukhov, and Conklin 2010; see reviews in Nathans, Oswald, and Nimon 2012; Johnson and Lebreton 2004; Grömping 2015), and progress in computing power increasingly allows for using these methods with even large data sets. Examples include variance decomposition, variable transformation, neural networks, and machine learning (Grömping 2015; Wei, Lu, and Song 2015). Hierarchical partitioning (HP) (Chevan and Sutherland 1991) is a method of variance decomposition based on measuring goodness of fit for all model subsets and measuring the independent contribution of each predictor (Mac Nally 2002; Lai et al. 2022). The random forests approach (RF) is one of the best machine‐learning algorithms for variable‐importance assessment, and RF can be interpreted as a variance decomposition method in a broad sense (Grömping 2015). RF can identify nonlinear relationships between the dependent and predictor variables, handle large numbers of variables with relatively small numbers of observations (Strobl et al. 2008), and can be effective in identifying relevant variables in high‐dimensional problems with highly correlated and interacting predictors, such as those commonly encountered in nature (Liu et al. 2021).

The goal of our study is to compare three statistical approaches that may provide contrasting, complementary perspectives on the variable importance of ecological predictors. Despite the large number of variable importance methods available (Wei, Lu, and Song 2015), we selected RF as a machine‐learning method, and HP as a case of variance decomposition method because they both have been widely used across various scientific fields and have demonstrated high prediction accuracy. Both methods can also address two important properties of the data: RF allows for non‐linear relationships, and both methods can separate the independent variance explained by predictors from conjoint explained variance. We used the important case of the worldwide distribution of alien species richness and predictors. Identifying the main drivers that shape the biological success and occurrence of alien species has become a challenging endeavor in contemporary ecology (Xu et al. 2024), particularly for the economic costs associated to the establishment of alien species out of their native distribution (Diagne et al. 2021). We compared the results of measures of variable importance (HP and RF) with the standard statistical regression methods generally used in ecology and evolution, namely, beta weights (Table S1). We also aimed to identify issues that are likely to apply to many other biological and scientific questions.

## 2 Materials and Methods

### 2.1 Data Collection

We used the comprehensive dataset provided by Dawson et al. (2017) (available at https://zenodo.org/records/556393#.WPjH08a1s2w) on the worldwide distribution of alien species richness of eight taxonomic groups and potential environmental and human socioeconomic predictors. The dataset, which consisted of amphibians, ants, birds, mammals, vascular plants, reptiles, spiders, and freshwater fishes, was curated by Dawson et al. (2017) to fit within the Biodiversity Information Standards (TDWG) geographic system (Brummit 2021). The set includes inventories of established alien species in 609 TDWG level 4 regions, which is the finest spatial scale from TDWG and mostly corresponds to countries or states and provinces within larger countries, and major islands and archipelagos. The total number of alien species in each region and global data coverage was compiled for each taxonomic group (Dawson et al. 2017). Dawson et al. (2017) is a high‐quality dataset because it provides information on the geographical area and sampling effort, which is important because alien species richness almost certainly depends on the size of the region and sampling intensity. Moreover, for amphibians, birds, vascular plants, ants, and mammals, survey completeness is also considered to represent the degree to which the documented species in a particular region (or grid cell) reflects the actual biota of that area (see further details in Dawson et al. 2017). Global data coverage for regions was highest for birds and mammals (609 regions), followed by vascular plants (449 regions, 82% of global ice‐free terrestrial area), ants, freshwater fishes, spiders and least for amphibians and reptiles (311 and 310 regions, 48% and 47% of area, respectively). Given that species richness values per region differed greatly among taxonomic groups, alien species richness was scaled from 0 to 1 for each taxonomic group (see further details in Dawson et al. 2017). Last, we removed islands following Winter et al. (2010) because invasion processes almost certainly differ on islands from continental areas (Moser et al. 2018; Essl et al. 2019).

The dataset of Dawson et al. (2017) includes a suite of environmental and socioeoconomic variables that may relate to the spatial patterns of alien species. We used the same environmental variables as did Dawson et al. (2017) without adding more variables because our objective was to evaluate variable importance among different statistical techniques, using a comprehensive, standard ecological dataset. Therefore, gross domestic product per capita (GDPc) (US dollars) was available for each TDWG level 4 region as the average of estimated values in 1 km^2^ grid cells, using estimates from nighttime light in satellite data (Ghosh et al. 2010). Although nighttime satellite data are a coarse proxy of actual economic data, particularly for those countries with large populations and relatively low lighting (e.g., China, India), they can detect low‐intense light areas and provide a larger extent of the global economy (Ghosh et al. 2010). Human population density (HPD) in the year 2000 was calculated in a similar manner from 1 km^2^ grid‐cell values obtained from the Global Rural Urban Mapping Project (GRUMP; http://sedac.ciesin.columbia.edu/data/set/grump‐v1‐population‐density). From the data provided by Dawson et al. (2017), we also computed absolute measures for each region, namely: gross domestic product (GDP) was estimated by multiplying the gross domestic product per capita (GDPc) by the population density (in 2000) and geographical area (km^2^) for each region; and (total) human population (in 2000) was estimated by multiplying the population density and geographical area of each region. The two relative (GDPc and HPD) and absolute (GDP and HP) measures inform about different properties: GDPc is more the economic development or standard of living (i.e., also accumulated human impacts) compared to GDP, which is more overall economic activity (Taylor and Irwin 2004; see world maps in Figure S2). GDP and GDPc were not highly correlated (Pearson's *r* = 0.075; *p* = 0.101), while HP and HPD were moderately correlated (Pearson's *r* = 0.533; *p* < 0.001).

In Dawson et al. (2017), climate data such as mean annual temperature (MAT) and mean annual precipitation (MAP) were obtained at 1‐min resolution from WORLDCLIM (www.wordclim.org; mean annual temperature = BIO1 and mean annual precipitation = BIO12 from the bioclim variables), and averages were calculated for each TDWG level 4 region.

### 2.2 Statistical Analyses

We used linear mixed models (LMM), as in Dawson et al. (2017), which arguably is the commonest approach used in ecology and evolution (Table S1). We used RF and HP as alternative techniques to estimate measures of relative variable importance. The three techniques followed designs similar to the mixed models in Dawson et al. (2017) to facilitate comparisons among methods (Figure S3). For the HP and LMM (but not in RF because the outcome is invariant to log transformation; Breiman 2001), we log‐transformed the region area, and the relative and absolute measures of economy and population, and we used a square‐root transformation for precipitation to satisfy assumptions of linearity, normality of residuals and homoscedasticity and to have the same LMMs as in Dawson et al. (2017).

#### 2.2.1 Linear Mixed Models

We used LMM, with the same design as in Dawson et al. (2017), as a baseline for comparison and as an example of the standard analysis in ecology and evolution (Table S1). The fixed effects were area, sampling effort (average % native species completeness), gross domestic product per capita (GDPc), human population density (HPD), mean annual temperature, mean annual precipitation, and whether the region was coastal or landlocked. The random effects consisted of the (subcontinental) TDWG Level 2 regions, nested within TDWG continents (random intercepts only). We inspected corrected Akaike's Information Criterion for all full models, and all models nested within them, to identify the set of models with the lowest‐AIC values, which were selected for inference. Given that the AIC criterion does not inform about the goodness of fit of the model (Mac Nally et al. 2018), we also calculated the marginal *R* ^2^ (accounting for fixed effects) and conditional *R* ^2^ (accounting for both fixed and random effects) following Nakagawa and Schielzeth (2013). Sampling effort was not included in models explaining alien fish, reptile, and spider richness because data on native species inventory completeness were not available for these taxonomic groups (Dawson et al. 2017). We used the R package *nlme* (Pinheiro et al. 2024) to compute the linear mixed models and *MuMIn* (Bartoń 2022) to obtain the marginal and conditional *R* ^2^. Last, we used Pearson correlations and a principal component analysis, using R packages *FactoMineR* (Lê, Josse, and Husson 2008) and *factoextra* (Kassambara and Mundt 2016) to examine the correlations among the predictors and for data visualization to further interpret the results (see correlation and principal component analyses in Figures S4, S5, respectively).

#### 2.2.2 Random Forests (RF)

The goal of RF is to create a set of classification rules (tree branches) from the predictors included in a training data (70% of the total dataset per each taxonomic group) (Breiman 2001). RF combines multiple decision trees to improve classification accuracy and reduce overfitting. Each tree is built on a different subset of the data (created by bootstrapping) and is trained on a random subset of predictors at each split. The classification rules are built by recursive binary partitioning of the training data that split into two nodes from two regions that are increasingly homogenous with respect to the classification variable (Cutler et al. 2007). This split is selected according to the Gini index, which measures the quality of its contribution to the classification (Therneau and Atkinson 1997). Data samples are generated by a bootstrap technique (Cutler et al. 2007). Individuals (in our case specific TDWG level 4 regions) present in a bootstrap sample are referred to as ‘in‐bag’ data, whereas the remaining individuals form the ‘out‐of‐bag’ (OOB) data. A non‐pruned classification tree is built from each bootstrap sample with the RF. We previously tuned the RF to optimize its performance and enhance its predictive capabilities using the iterative random forest (iRF) algorithm (Basu et al. 2018). iRF searches for the optimal number of variables to randomly sample to be as candidates at each split (Basu et al. 2018). We then applied RF to each taxonomic group with 10,000 trees and considering the optimal number of variables (‘mtry’ parameter) found with the iRF algorithm. The typical variable importance in RF measures the impact of a predictor for explaining the response variable without taking any other predictors into account, that is, the marginal effects (here referred as ‘unconditional variable importance’), whereas measures of conditional permutation importance (here referred as ‘conditional variable importance’) quantify the impact of predictors after controlling for the other predictors in the model (Debeer and Strobl 2020). The threshold value in the conditional reflects the degree of tuning to make the permutation conditional and was set at a standard threshold of 0.95, following Debeer and Strobl (2020). RF analysis was performed with the packages *tuneRF* for tuning the RF (Basu et al. 2018), *randomForest* (Liaw and Wiener 2002), and *permimp* for conditional variable importance (Debeer and Strobl 2020) in the software R (R Core Team 2023).

#### 2.2.3 Hierarchical Partitioning (HP)

We conducted HP analyses to evaluate the relative importance of each predictor together with the shared variation (Chevan and Sutherland 1991). HP can overcome problems related to model‐selection procedures, which may fail to provide a valid means for ranking the relative importance of the predictors (Whittingham et al. 2006; Mundry and Nunn 2009). It also has the advantage to overcome challenges associated with multicollinearity since HP calculates the unique contribution of each variable, while controlling for shared variation. HP uses all combinations of predictors (2^*N*^ for *N* predictors) to determine the amounts of variation explained by each predictor (Lai et al. 2022). This is achieved by calculating the individual shared percentage of variation explained by the model (semi‐partial *R* ^2^) (Chevan and Sutherland 1991). If a predictor had a negative semi‐partial *R* ^2^ due to the strong and complex correlation among predictors (Peres‐Neto et al. 2006), we set the value to 0. We evaluated the statistical significance in HP analyses based on 999 permutations (Lai et al. 2022). We applied HP for each taxonomic group with only the relative measures (HPD, and GDPc), only the absolute measures (HP and GDP), and both. We used the R package *rdacca.hp* to estimate the relative importance of each predictor in multivariate models through canonical analysis, without limiting the number of predictors (Lai et al. 2022).

## 3 Results

**FIGURE 2:**
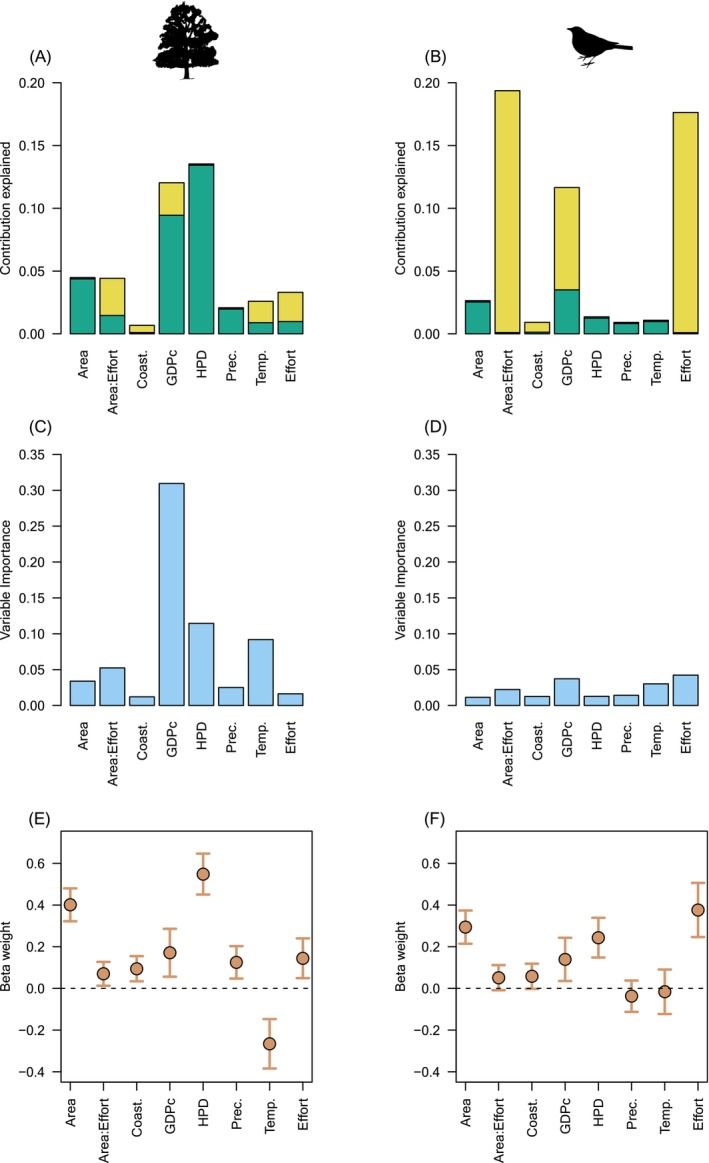
Variable importance of the predictors of regional alien species richness of plants (A, C, and E; *n* = 347 regions) and birds (B, D, and F; *n* = 423 regions) using three statistical techniques: Hierarchical partitioning (A and B), random forests (C and D), and mixed linear models (E and F) (results for the other taxonomic groups are shown in Figures 3, 4, 5). For hierarchical partitioning, the individual (green) and shared (yellow) contributions are shown. The conditional (threshold = 0.95) variable importance of all predictors of random forests is shown in light blue bars. Mixed models refer to the beta weights (brown circles) and their 95% confidence interval. For the HP and LMM, region area, and the relative and absolute measures of economy and population were log‐transformed, and precipitation was square‐root transformed. GDPc, gross domestic product per capita; and HPD, human population density.

**FIGURE 3:**
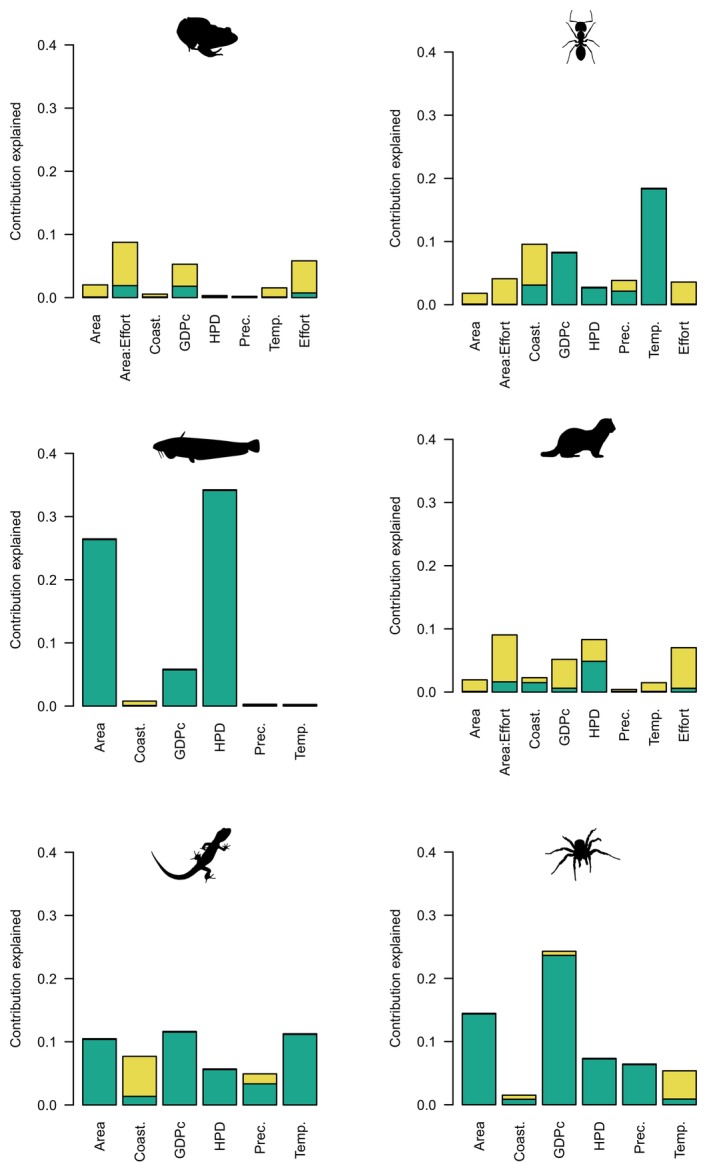
Variable importance of the predictors of regional alien species richness worldwide for taxonomic groups using hierarchical partitioning analyses. The individual (green) and shared (yellow) contributions are shown. The selected predictors are the same ones used by Dawson et al. (2017; but note that islands have been excluded here) to facilitate comparison with traditional regression analyses. GDPc, gross domestic product per capita; and HPD, human population density.

**FIGURE 4:**
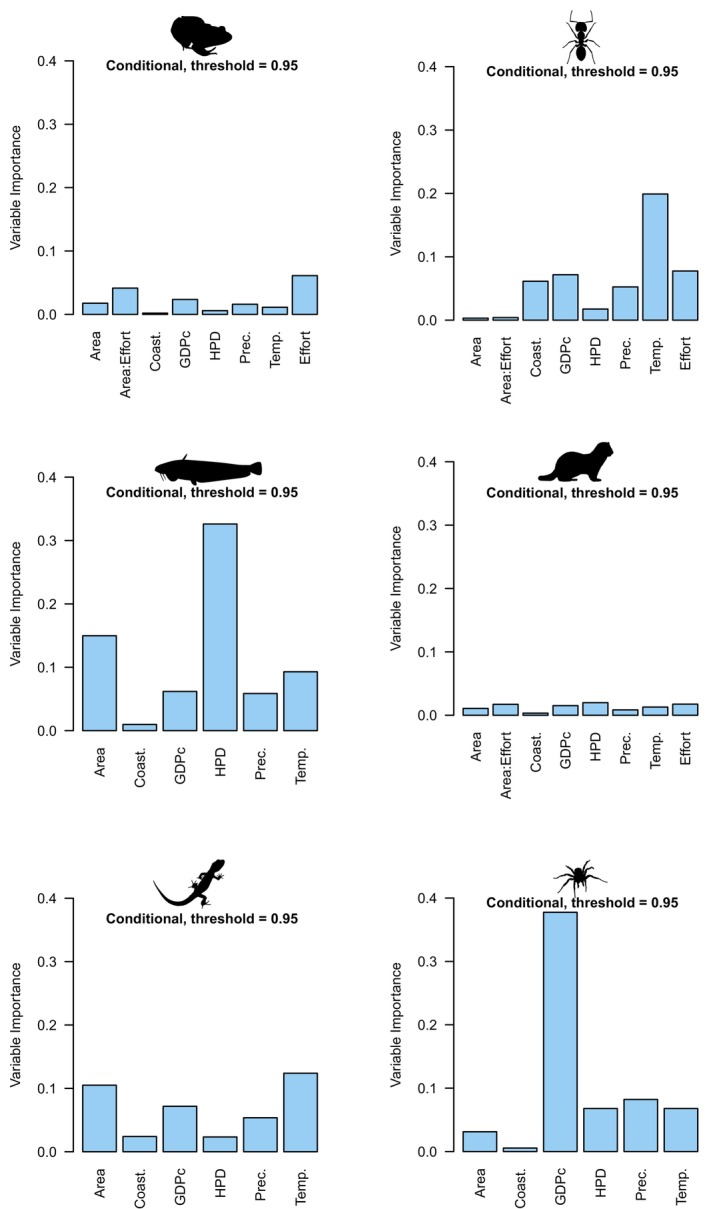
Variable importance of the predictors of regional alien species richness worldwide using random forests. The conditional (threshold = 0.95) variable importance is shown in light blue bars. The selected predictors are the same ones used by Dawson et al. (2017; but note that islands have been excluded here) to facilitate comparison with traditional regression analyses. GDPc, gross domestic product per capita; and HPD, human population density.

**FIGURE 5:**
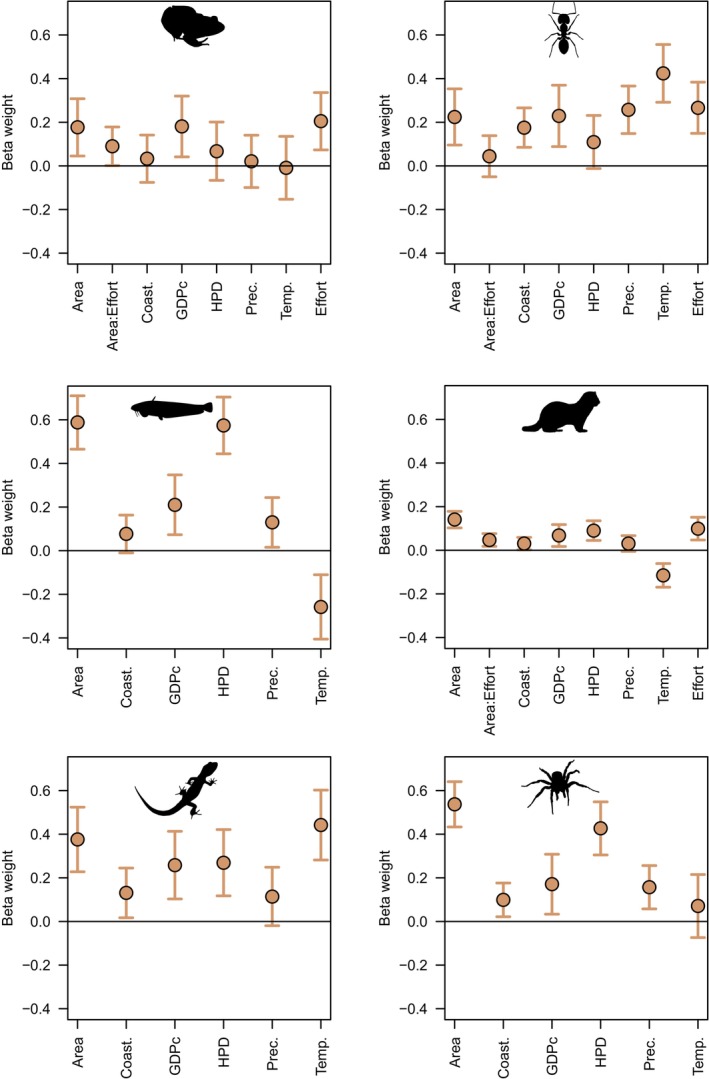
Beta weights (and 95% confidence intervals) of the predictors of regional alien species richness worldwide using mixed models. The selected predictors are the same ones used by Dawson et al. (2017; but note that islands have been excluded here) to facilitate comparisons. Note that there was no information available of sampling effort for fish, reptiles, and spiders. GDPc, gross domestic product per capita; and HPD, human population density.

Two very different analytical techniques (HP and RF) to measure variable importance, but which each deal explicitly with variable intercorrelations, generally provided similar rankings of predictors (Table 1, Figures 1D, 2A, 3, 4, and Figure S7), in contrast to regression coefficients of LMM (Table 1, Figures 1F, 2E, 5). HP and RF suggested that GDPc and HPD were the most important predictors for plants (Figures 1C, 2A), whereas LMM suggested that HPD and area were the most influential (Figure 2E). For birds, the variable importance techniques suggested that GDPc was the most important predictor, after controlling for sampling effort (Figure 2B); by contrast, LMM suggested that area and HPD were more influential (Figure 2F). The results for other taxa were similar: LMM tended to highlight the effects of area, HPD, and temperature whereas the other techniques attributed more importance to GDPc (Table S2, Figures 3, 4, 5, and Figure S7).

**TABLE 1:** Comparison between the results of linear mixed models and of hierarchical partitioning in explaining the alien species richness of plants and birds.

Group	Variable	Mixed model beta weights	*p*	Hierarchical partitioning independent explained variance	*p*
Plants (0.288, 0.810)	Area	**0.401**	***	0.041	**0.001**
	Area × effort	**0.070**	**0.017**	**0.044**	**0.001**
	Coast.	**0.094**	**0.002**	0.005	0.113
	GDPc	**0.171**	**0.004**	**0.120**	**0.001**
	HPD	**0.548**	***	**0.082**	**0.001**
	Precipitation	**0.125**	**0.002**	**0.013**	**0.027**
	Temperature	**−0.266**	***	**0.026**	**0.004**
	Effort	**0.144**	**0.003**	**0.033**	**0.002**
	TOTAL			0.364	
Birds (0.343, 0.650)	Area	**0.294**	***	**0.024**	**0.002**
	Area × effort	0.051	0.095	**0.193**	**0.001**
	Coast.	0.058	0.059	**0.009**	**0.042**
	GDPc	**0.139**	**0.009**	**0.116**	**0.001**
	HPD	**0.243**	***	0.005	0.083
	Precipitation	−0.037	0.330	**0.009**	**0.031**
	Temperature	−0.016	0.764	**0.010**	**0.031**
	Effort	**0.376**	***	**0.175**	**0.001**
	TOTAL			0.541	

*Note:* The variables incorporated in the model were the same as Dawson et al. (2017), but islands have been excluded from the analyses here. Significant *p* values (*p* < 0.05) are bolded. See the results for the other taxonomic groups in the Supporting Information. The marginal and conditional *R* ^2^ for mixed models are given in parentheses; the equivalent “total” for hierarchical partitioning is also given.

Abbreviations: GDPc, gross domestic product per capita; and HPD, human population density.

*** Indicates *p* < 0.001.

Results for HP also showed that much of the variation explained was shared among predictors, but this differed among taxa; the individual contribution was generally higher for temperature and precipitation and lower for socioeconomic variables, latitude, and area (Figures 2, 3 and Figure S7). The correlations among predictors (Figures S4, S5) were relatively low (|*r*| always < 0.41), which and helped us to understand these unique contributions and the contrasting results above. Most predictors were intercorrelated but coastal (vs landlocked mainland) region, precipitation, HPD, and sampling effort had the weakest correlations and GDPc the strongest with the other predictors (Figures S4, S5). In agreement, beta weights were more different from zero for HPD and sampling effort (and secondarily, precipitation) in contrast to the two other techniques, whereas GDPc had beta weights closer to zero because it was more correlated to many predictors, despite having high relative importance and unique contributions (Figures 2, 5).

**FIGURE 6:**
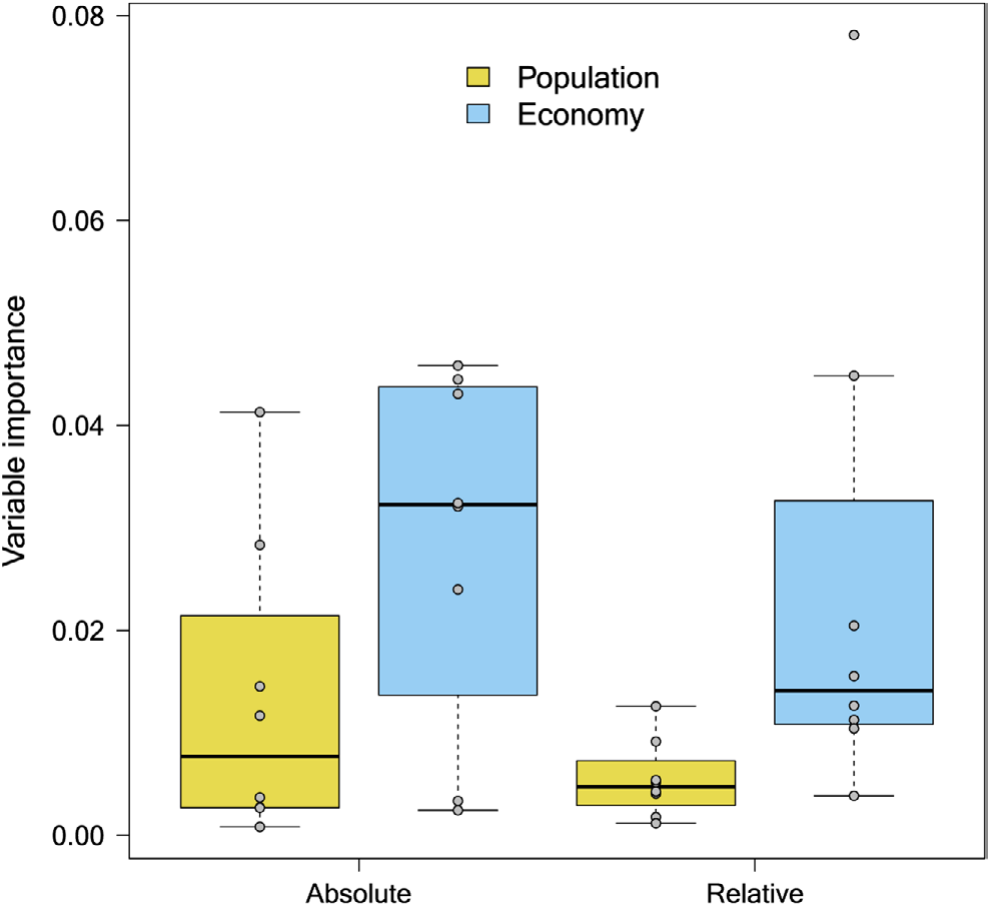
Box‐plot of variable importance (conditional, threshold = 0.95) of the population and economic predictors in explaining regional alien species richness for all taxonomic groups. The variable importance for the random forests of the seven taxonomic groups is shown, separately for absolute (GDP, gross domestic product; and HP, human population) and relative (GDPc, gross domestic product per capita; and HPD, human population density.) predictors.

HP and RF analyses including both absolute and relative measures (Figure 6, Figures S7–S10) suggested that human economic indicators (GDP and GDPc) are generally much more important than human population size and density to explain alien species richness. Specifically, HP indicated that, except for mammals, the total GDP of a region is more important than its human population and that GPDc is more important than human population density (Figure 6, Figure S7). HP also showed that absolute measures (i.e., GDP and HP) are more important than their relative counterparts (GDPc and HPD), except for mammals and birds (Figure S7). Conditional variable importance of random forests, which are not constrained by collinearity issues, suggested that GDPc is more important than GDP to explain alien species richness (Figure S9).

Although some independent contributions were small, most were significant for both LMM and HP analyses (Table 1 and Table S2). The orderings of conditional (Figures 2, 4, S8, and S10) and unconditional (Figures S6, S9) RF measures of variable importance were similar. Climatic features differed markedly among taxa; increasing temperature is associated with greater alien richness of ants, amphibians, reptiles, birds, mammals, but has the opposite association with plants and fish; the richness of alien ants, plants, and spiders is positively related to precipitation but is not so for other taxa. Socioeconomic variables were more consistent and always displayed positive associations with richness (Figure S10).

## 4 Discussion

### 4.1 Quantification of Variable Importance

Ecological and evolutionary research deals with a large array of interacting factors influencing the phenomenon of interest. Identifying the relative importance of predictors is a challenging task given the presence of interactions and collinearity and that variables do not operate in isolation. In the present study, we explored three methods to quantify the relative importance of the main drivers of regional alien species richness worldwide, including classical regression approaches (Dawson et al. 2017). We suggest that the two compared variable importance measures (RF and HP) delivered improved insights about the spatial patterns of alien species richness, and, in turn, on the most likely drivers. Specifically, our analysis revealed that economic drivers (both relative and absolute) were more important than population variables for alien species richness.

Although it is well known in the statistical literature (see references in the Introduction and Figure 1) that RF and HP can better reflect variable importance compared to partial regression coefficients, the latter remain the most widely used methods by ecologists for such purposes (Table S1). Due to progress in statistical methods, data availability, and computing power, machine‐learning methods have substantially increased in use among ecologists (Lucas 2020) but mostly for species‐distribution modeling because of the higher predictive power (Bradter et al. 2013). In addition to invasion biology, the adoption of RF and HP to identify variable importance should be extended to many other topics such as climate change or habitat fragmentation (Mac Nally 2000; Leng et al. 2008; Smith et al. 2009; Zheng 2018). The use of partial regression coefficients or their standardized versions (i.e., beta weights)—commonly employed indices to quantify and understand the ecological relationships between various explanatory variables and a response variable—should be treated with caution to unravel the variable importance of an ecological phenomenon, particularly when dealing with complex, nonlinear or high‐dimensional data. A clear example is Figure 2, where RF and HP identified GDPc and HPD as the most important factors for alien plant species and GDPc and sampling effort for alien bird species. In contrast, LMM highlighted HPD, region area, sampling effort, and environmental drivers such as temperature (Figure 2).

Our results also showed that the relative importance of predictors that are weakly intercorrelated is overestimated in linear regression, as has been inferred in previous works (Galipaud et al. 2014; Giam and Olden 2016; Lai et al. 2022). Similarly, traditional regression models will rarely be able to cope with situations requiring the inclusion of numerous explanatory variables (Breiman 2001). Despite the recognized shortcomings of (generalized) linear regression models, such approaches still are widely used in recent literature (Planque and Buffaz 2008; Yee, Yeung, and Cheng 2008; Bolker et al. 2009; Gompert and Buerkle 2009; Koper and Manseau 2009). Despite many variables being intercorrelated, our results with LMM still show a significant influence of the main drivers shaping the spatial patterns of alien species richness.

### 4.2 Consequences for Understanding Drivers of Biological Invasions

Increasing economic activities and human population exert a positive influence on the number of alien species among taxa (Taylor and Irwin 2004; Westphal et al. 2008). To the best of our knowledge, our study is the first to compare the variable importance of absolute and relative metrics of economic and human population. Relative socioeconomic variables (i.e., GDPc) provide insights into standards of livings (Pyšek et al. 2010; Moser et al. 2018), while absolute variables (i.e., GDP) offer information about the intensity of economic activities related to commercial trade, industrialization, and urbanization (Taylor and Irwin 2004; Leprieur et al. 2008). This distinction becomes apparent in Scandinavian countries, characterized by high values of GDPc alongside low human populations, whereas densely populated countries such as China or India exhibit high GDP but lower values of GDPc (Figure S2). These differences could be related to different introduction pathways, such as the pet trade, involving intentional introductions through the deliberate release of birds and mammals and being more important in countries with higher GDPc (Hulme et al. 2008) and overall commercial trade, more related to GDP and to unintentional introduction processes (Chapple, Simmonds, and Wong 2012). Our HP results (e.g., Figure S7) suggest that both absolute and relative measures play a role in alien richness, but that the former are more important and that future studies should carefully consider the difference between relative and absolute factors when investigating underlying drivers and predicting the distribution of alien species. Similarly, climate‐related variables (temperature and precipitation) had high individual contributions, reflecting their strong, independent influence on alien species. Our findings highlight the predominant role of socioeconomic variables in elucidating patterns of alien species compared to human population variables. Moreover, our results might be generalized to other ecosystems and taxa to some extent. While we identified repeated ecological patterns based on the selection of our drivers as being key in invasion research (Diagne et al. 2021; Xu et al. 2024), the variable importance of individual drivers may vary depending on the type and number of drivers included in the analyses.

### 4.3 Limitations and Future Research

The data used in the current study was delineated based on the best available information to cover multiple taxonomic groups and a global coverage. However, it also has some caveats. Drivers are characterized by coarse scale and general information, which may obscure fine‐scale ecological dynamics and specific spatial patterns of alien species richness. For instance, integrating high spatial resolution data, such as microclimatic variables or fine land use changes, can provide more nuanced insights into the drivers of alien species richness. In cases where such data are not readily accessible, researchers should consider developing methods to downscale coarse data or apply spatially‐explicit modeling techniques (e.g., satellite data) to bridge this gap.

Similarly, the interpretability of our economic and population drivers is not easy and can lead to a misunderstanding of the actual ecological processes. Economic and population density drivers serve as proxies for the underlying drivers of alien species, but these metrics are complex and might sometimes not be effective indicators. For instance, GDP captures the intensity of economic activities such as trade, industrialization, and urbanization but the movement of alien species across countries is often poorly regulated (Carlton and Ruiz 2015). GDPc may reflect standards of living and investment, but ignores local economies (e.g., tourist impact, housing). Similarly, HPD and HP are proxies for human‐mediated disturbance and propagule pressure. To complicate matters further, the relationships between these variables and alien species richness may vary among taxa and regions. GDPc may be more relevant in countries with intentional introductions through trade and the pet industry, while GDP could better explain unintentional introductions driven by high trade volumes.

Last but not least, some caution is needed because, as usual, we were unable to directly quantify other important invasion processes, such as propagule or colonization pressures (Simberloff 2009; Lockwood, Cassey, and Blackburn 2009). This suggests a potential overestimation of the impact of human population variables in analyses exploring factors influencing macroecological invasion patterns. The current state of invasive research lacks a comprehensive comparison among major groups of drivers, with only a few studies considering both environmental and economic factors (McKinney 2002; Pyšek et al. 2010). However, these studies did not conduct statistical analyses aimed at determining the net effects of individual variables and their relative importance.

## 5 Conclusions

Relying on the variable importance of predictors of ecological phenomena is intimately connected with management and policy. The statistical identification of the most influential predictors for a given phenomenon (e.g., invasive species success) enables better environmental policy‐making and conservation decisions. That is, in real‐world scenarios, where many drivers interact among them, finding alternative techniques to cope with issues of multicollinearity and model selection can help to identify the most important drivers shaping the spatial patterns of alien species. Integrating the evaluation of variable importance into statistical analyses can be crucial for interpreting results in ecological and evolutionary studies. Future research with RF and HP techniques, possibly among use of other modern developments, holds promise in integrating drivers from both coarse and fine scales, and variable importance measures can be applied to address other biodiversity questions, particularly those arising from the impacts of global change.

## Author Contributions

**Ignasi Arranz:** conceptualization (lead), formal analysis (lead), investigation (equal), writing – original draft (lead). **Ralph Mac Nally:** investigation (equal), validation (equal), writing – review and editing (equal). **Emili García‐Berthou:** conceptualization (lead), methodology (equal), supervision (equal), validation (equal), writing – review and editing (equal).

## Conflicts of Interest

The authors declare no conflicts of interest.

## Supporting information


Data S1.

## Data Availability

The invasive dataset is freely available from Dawson et al. (2017). It is also available at https://zenodo.org/records/556393#.WPjH08a1s2w.
