# Supplementary material for: Variable Importance Measures Suggest Paramount Influence of Human Economics on Alien‐Species Introductions
Source: Ecol Evol. 2025 Feb 12;15(2):e70965. doi: 10.1002/ece3.70965 (PMC11815206; doi:10.1002/ece3.70965)
Supplement: Supplementary file 1 — Data S1. [file ECE3-15-e70965-s001.docx]

**Supporting Information to “Variable importance measures suggest paramount influence of human economics on alien-species introductions”**

Ignasi Arranz, Ralph Mac Nally & Emili García-Berthou

**Fig. S1**. R script to reproduce the results of Figure 1.

#=================================#

# Script to reproduce the results of Fig. 1 of

# "Variable importance measures suggest paramount influence of economy in explaining alien introductions"

#

#=================================#

# Set the environment

rm(list=ls(all=TRUE))

# Load packages

packages <- c("corpcor", "MASS", "PerformanceAnalytics", "rdacca.hp", "randomForest","permimp","gmodels")

install.packages(setdiff(packages, rownames(installed.packages())))

library(corpcor) #make.positive.definite()

library(MASS) #function mvrnorm()

library(PerformanceAnalytics) #function checkData()

library(rdacca.hp) #function rdacca.hp()

library(randomForest) #function randomForest()

library(permimp) #function permimp()

library(gmodels) #function ci()

# Create data

# data and first part of the script modified from Ray-Mukherjee et al. (2014) doi: 10.1111/2041-210X.12166

covm <- c(1.00, 0.30, 0.00, 0.20, 0.20,

0.30, 1.00, 0.10, 0.10, 0.10,

0.00, 0.10, 1.00, 0.50, 0.50,

0.20, 0.10, 0.50, 1.00, 0.50,

0.20, 0.10, 0.50, 0.50, 1.00)

covm <- matrix(covm,5,5)

covm <- make.positive.definite(covm)

varlist <- c("Y","X1","X2","X3","X4")

dimnames(covm) <- list(varlist,varlist)

set.seed(1)

data1 <- mvrnorm(n=200,rep(0,5), covm, empirical=TRUE)

data1 <- data.frame(data1)

# Figure 1A: Univariate correlations

chart.Correlation(data1,hist=F,method = "pearson",cex.labels =3)

# The warnings are due to problems in this function but do not affect the results

# see https://quant.stackexchange.com/questions/16114/chart-correlation-warnings for further details

text(x = 0.2, y = 0.95, labels = "(A) Univariate correlations", xpd = NA,cex=1.5)

# Figure 1B: Regression

lmOut <- lm(Y~X1+X2+X3+X4,data1)

df <- as.data.frame(ci(lmOut))

df <- df[-1,] #Remove Intercept

plot(df$Estimate~c(1:length(df$Estimate)),

ylim = c(min(df$`CI lower`), max(df$`CI upper`)),

xaxt = "n",

xlab="",

main="",

pch=21,

col="white",

bg="white",

las=1,

ylab = "Beta weight",

cex.lab=1.5)

arrows(x0 = c(1:dim(df)[1]),

y0 = df$`CI lower`,

x1 = c(1:dim(df)[1]),

y1 = df$`CI upper`,

angle = 90, code = 3, length = 0.05, col = "#D2986B",lwd=2)

points(df$Estimate,cex=2,pch=21, bg="#D2986B",col="black")

axis(side = 1, at = 1:dim(df)[1], labels = c("X1","X2","X3","X4"), tick = T,las=1,cex.axis=1.25)

abline(h=0,lty=2)

text(x = 1, y = 0.50, labels = "(B) Multiple regression", xpd = NA,cex=1.5)

# Figure 1C: Hierarchichal Partitioning

spe <- data1$Y

env <- data.frame(data1[,c(2:5)])

spe.hp <- rdacca.hp(spe,env,method="RDA", type="adjR2",var.part=T) #rdacca.hp for variation and hierarchical partitioning

vector.indi <- spe.hp$Hier.part[,"Unique"]

vector.share <- spe.hp$Hier.part[,"Average.share"]

ind <- data.frame(factor = paste("X",c(1:4),sep = ""),value = vector.indi, category = "ind")

rownames(ind) <- NULL

share <- data.frame(factor = paste("X",c(1:4),sep = ""),value = vector.share, category = "shar")

rownames(share) <- NULL

df <- rbind(ind,share)

totals <- tapply(df$value, list(df$category, df$factor), sum)

colors <- c("#1BA68D","#E7DA4F")

barplot(totals,

col = c(colors),

xlab = "", ylab = "Contribution explained",

cex.lab=1.5,

cex.names=1.25,

cex.axis=1,

cex.main=1,

ylim=c(0,0.1),las=1,

names=paste("X",c(1:4),sep = ""))

text(x = 1, y = 0.11, labels = "(C) Hierarchical Partitioning", xpd = NA,cex=1.5)

# Figure 1D - 1E: Random Forest

RF.model_daw <- randomForest(Y~X1+X2+X3+X4,data=data1,importance = TRUE)

imp986b <- permimp(RF.model_daw, conditional = TRUE, threshold = 0.95, do_check = FALSE)

barplot(imp986b$values, las=1,

ylab="Variable Importance",

cex.lab=1.5,

cex.names=1.25,

cex.axis=1,

ylim=c(0,0.15),col="#98CEF3",

names=paste("X",c(1:4),sep = ""))

text(x = 2, y = 0.16, labels = "(D) Random Forest (Conditional, threshold = 0.95)", xpd = NA,cex=1.5)

imp987a <- permimp(RF.model_daw, do_check = FALSE)

barplot(abs(imp987a$values), las=1,

ylab="Variable Importance",

cex.lab=1.5,

cex.names=1.25,

cex.axis=1,

ylim=c(0,0.15),col="#98CEF3",

names=paste("X",c(1:4),sep = ""))

text(x = 1, y = 0.16, labels = "(E) Random Forest (unconditional)", xpd = NA,cex=1.5)

### End of the script ###

**Fig. S2**. Global distribution of the macroeconomic and demographic variables. Absolute (Gross Domestic Product, GDP; and Human Population, HP) and relative (Gross Domestic Product per capita, GDPc; and Human Population Density, HPD) measures are shown. Grey areas correspond to regions with no information available.

**Fig S3.** Flowchart summarizing the main steps in the data analyses with the three statistical techniques.


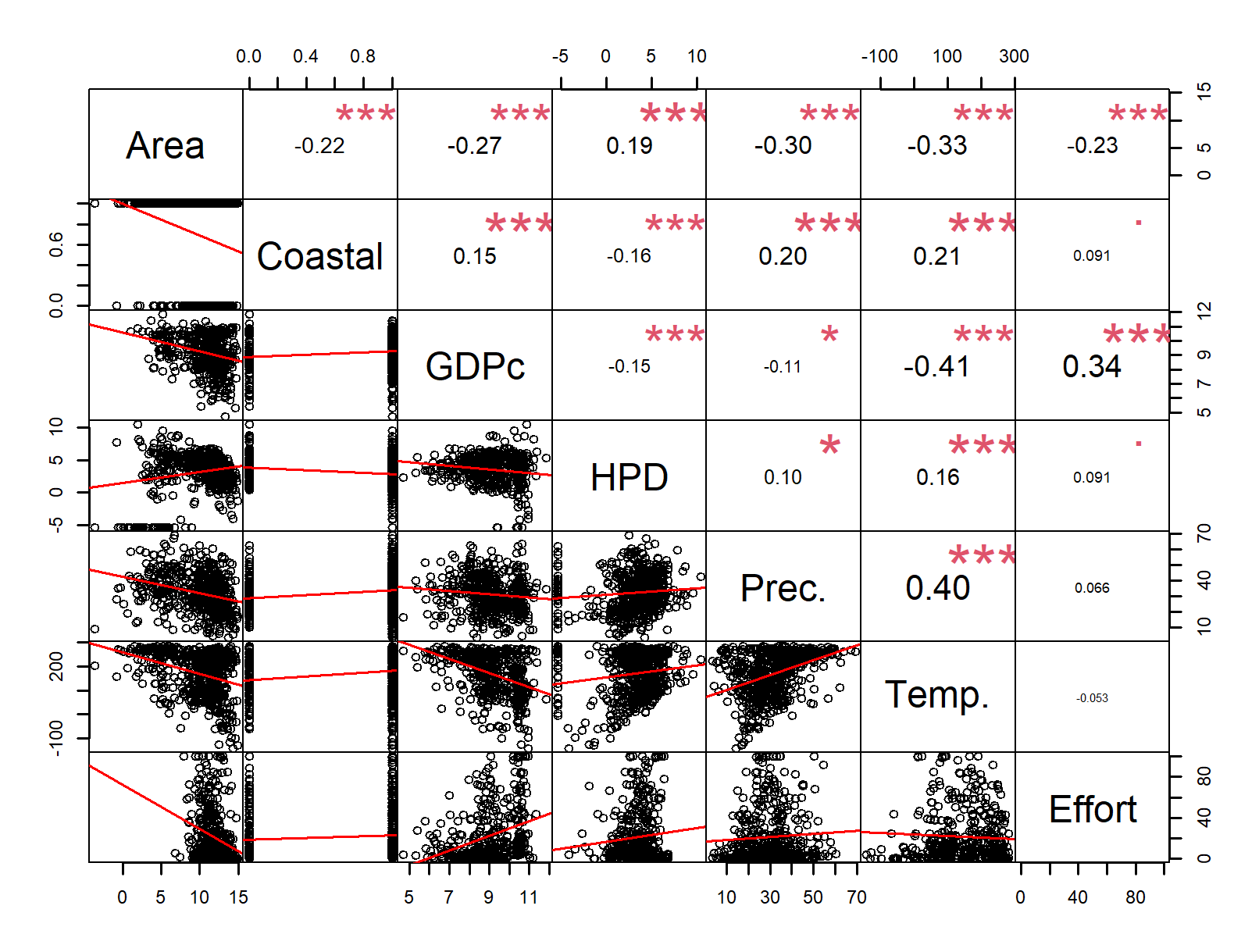


**Fig. S4**. Pairwise relationships between the predictors of alien richness, using the dataset of Dawson et al. (2017). Below the diagonal, the bivariate scatterplots with the linear regression function; above the diagonal, the Pearson correlation coefficients with significant coefficients levels (■, *P* < 0.10; *, *P* < 0.05; **, *P* < 0.01; ***, *P* < 0.001). Following Dawson et al. (2017), we log-transformed the region area, and the relative and absolute measures of economy and population, and we used a square root transformation for precipitation to meet the assumptions of normality and homoscedasticity. GDPc: Gross Domestic Product per capita, and HPD: Human Population Density.

**Fig. S5**. Principal component analysis (PCA) of the predictors from Dawson et al. (2017). (A) First two PCA axes summarizing the correlations among predictors, with their contributions by color. (B) Contributions of the predictors to the first two PCA axes. GDPc: Gross Domestic Product per capita, and HPD: Human Population Density.

**Fig. S6.** Assessing the importance of predictors on the spatial patterns of regional alien species richness with Random Forests. Unconditional variable importance of all predictors of random forests is shown in light blue bars. The selected predictors are the same to the ones used by Dawson et al. (2017; but note that islands have been excluded here) to facilitate result comparison with traditional regression analyses. GDPc: Gross Domestic Product per capita, and HPD: Human Population Density.

**Fig. S7.** Variable importance of the predictors of regional alien species richness in the Hierarchical Partitioning analysis. The individual (green) and shared (yellow) contributions are shown. The included predictors encompass latitude, longitude and both relative and absolute measures of economy and population. GDPc: Gross Domestic Product per capita, HPD: Human Population Density, GDP: Gross Domestic Product, and HP: Human Population.

**Fig. S8.** Variable importance of the predictors of regional alien species richness in the Random Forests (conditional, threshold = 0.95). The light blue bars represent the variable importance of each predictor. The included predictors encompass both relative and absolute measures of economic and demographic processes. GDPc: Gross Domestic Product per capita, HPD: Human Population Density, GDP: Gross Domestic Product, and HP: Human Population.

**Fig. S9.** Variable importance of the predictors of regional alien species richness in the Random Forests (unconditional). The light blue bars represent the variable importance of each predictor. The included predictors encompass both relative and absolute measures of economic and demographic processes. GDPc: Gross Domestic Product per capita, HPD: Human Population Density, GDP: Gross Domestic Product, and HP: Human Population.

**Fig. S10.** Economic and population drivers shaping regional alien species richness in the Random Forests (Conditional, threshold =0.05). Color lines represent the taxonomic group. GDPc: Gross Domestic Product per capita, HPD: Human Population Density, GDP: Gross Domestic Product, and HP: Human Population.

**Table S1**. Number of articles published in 2023 in the British Ecological Society journals (except for *Methods in Ecology and Evolution*) using the three statistical techniques discussed. The search included statistical techniques with quotation marks to get an exact match for the name. Bold numbers represent the statistical technique most common in each journal. *Ecol. solut. evid.: Ecological Solutions and Evidence. Ecol. Evol.: Ecology and Evolution. Funct. Ecol.: Functional Ecology. J. Anim. Ecol.: Journal of Animal Ecology. J. Appl. Ecol.: Journal of Applied Ecology. J. Ecol.: Journal of Ecology. People nat.: People and Nature.*

| Statistical technique | *Ecol. solut. evid.* | *Ecol. Evol* | *Funct. Ecol.* | *J. Anim. Ecol.* | *J. Appl. Ecol.* | *J. Ecol.* | *People nat.* |
| --- | --- | --- | --- | --- | --- | --- | --- |
| "mixed model" | **6** | **102** | **31** | **35** | **25** | **39** | 2 |
| "generalised linear model" or  "generalized linear model" | 4 | 82 | 13 | 7 | 21 | 22 | **5** |
| "multiple linear regression" | 0 | 14 | 7 | 1 | 9 | 19 | 2 |
| "random forest" | 1 | 40 | 17 | 4 | 16 | 21 | 3 |
| "hierarchical partitioning" | 0 | 5 | 3 | 1 | 1 | 1 | 1 |

**Table S2**. Comparison between the results of linear mixed models and of hierarchical partitioning in explaining the alien richness of the remaining taxonomic groups. The variables incorporated in the model were the same as Dawson et al. (2017). Significant *P* values (*P* < 0.05) are bolded. *** indicates *P* < 0.001. The marginal and conditional *R*^2^ for mixed models are given in parentheses; the equivalent “total” for hierarchical partitioning is also given. GDPc: Gross Domestic Product per capita, and HPD: Human Population Density.

| Group | Variable | Mixed model  Beta weights | *P* | Hierarchical partitioning  Variable importance | *P* |
| --- | --- | --- | --- | --- | --- |
| Amphibians | Area | **0.177** | **0.009** | **0.015** | **0.045** |
| (0.221, 0.422) | Area × Effort | **0.090** | **0.046** | **0.088** | **0.001** |
|  | Coast. | 0.033 | 0.552 | 0.001 | 0.268 |
|  | GDPc | **0.181** | **0.011** | **0.053** | **0.002** |
|  | HPD | 0.067 | 0.322 | 0.000 | 0.311 |
|  | Precipitation | 0.021 | 0.733 | -0.001 | 0.330 |
|  | Temperature | -0.009 | 0.902 | 0.011 | 0.083 |
|  | Effort | **0.205** | **0.002** | **0.058** | **0.002** |
|  | TOTAL |  |  | 0.225 |  |
| Ants | Area | **0.224** | **0.001** | **0.015** | **0.035** |
| (0.403, 0.666) | Area × Effort | 0.044 | 0.355 | **0.041** | **0.002** |
|  | Coast. | **0.175** | ******* | **0.096** | **0.001** |
|  | GDPc | **0.229** | **0.002** | **0.061** | **0.001** |
|  | HPD | 0.109 | 0.078 | **0.013** | **0.035** |
|  | Precipitation | **0.257** | ******* | **0.038** | **0.004** |
|  | Temperature | **0.424** | ******* | **0.180** | **0.001** |
|  | Effort | **0.266** | ******* | **0.033** | **0.005** |
|  | TOTAL |  |  | 0.477 |  |
| Fish | Area | **0.588** | ******* | **0.137** | **0.001** |
| (0.308, 0.614) | Coast. | 0.077 | 0.083 | 0.005 | 0.138 |
|  | GDPc | **0.210** | **0.003** | **0.027** | **0.001** |
|  | HPD | **0.574** | ******* | **0.219** | **0.001** |
|  | Precipitation | **0.130** | **0.026** | 0.002 | 0.184 |
|  | Temperature | **-0.258** | **0.001** | 0.000 | 0.322 |
|  | TOTAL |  |  | 0.390 |  |
| Mammals | Area | 0.141 | ******* | **0.017** | **0.013** |
| (0.170, 0.801) | Area × Effort | 0.047 | **0.002** | **0.090** | **0.001** |
|  | Coast. | 0.031 | **0.035** | **0.023** | **0.002** |
|  | GDPc | **0.068** | **0.008** | **0.052** | **0.001** |
|  | HPD | **0.090** | ******* | **0.083** | **0.001** |
|  | Precipitation | 0.031 | 0.092 | 0.001 | 0.208 |
|  | Temperature | **-0.115** | ******* | 0.013 | **0.014** |
|  | Effort | **0.099** | ******* | **0.070** | **0.001** |
|  | TOTAL |  |  | 0.349 |  |
| Reptiles | Area | **0.376** | ******* | **0.062** | **0.001** |
| (0.338, 0.558) | Coast. | **0.131** | **0.025** | **0.077** | **0.001** |
|  | GDPc | **0.258** | **0.001** | **0.058** | **0.003** |
|  | HPD | **0.269** | **0.001** | **0.029** | **0.014** |
|  | Precipitation | 0.114 | 0.094 | **0.049** | **0.002** |
|  | Temperature | **0.442** | ******* | **0.091** | **0.001** |
|  | TOTAL |  |  | 0.366 |  |
| Spiders | Area | **0.537** | ******* | **0.069** | **0.001** |
| (0.192, 0.787) | Coast. | **0.099** | **0.012** | **0.015** | **0.031** |
|  | GDPc | **0.171** | **0.015** | **0.243** | **0.001** |
|  | HPD | **0.427** | ******* | **0.030** | **0.003** |
|  | Precipitation | **0.157** | **0.002** | **0.042** | **0.001** |
|  | Temperature | 0.071 | 0.337 | **0.054** | **0.001** |
|  | TOTAL |  |  | 0.453 |  |
